# In Silico Analysis of Selected Mikania Constituents As Human HMG-CoA Reductase, Human Inducible Nitric Oxide Synthase, and Human Squalene Synthase Inhibitory Agents

**DOI:** 10.7759/cureus.55110

**Published:** 2024-02-27

**Authors:** Sri Tharany Vahsh Vijayakumar, Radhakrishnan Narayanaswamy, Vasantha-Srinivasan Prabhakaran

**Affiliations:** 1 Biochemistry, Saveetha Medical College and Hospital, Saveetha Institute of Medical and Technical Sciences (Deemed to be University), Chennai, IND; 2 Bioinformatics, Saveetha School of Engineering, Saveetha Institute of Medical and Technical Sciences (Deemed to be University), Chennai, IND

**Keywords:** human squalene synthase (hsqs), human inducible nitric oxide synthase (hinos), human hmg coa reductase (hhmgr), docking, good health and well-being, mikania

## Abstract

Background

Numerous pharmacological activities have been reportedin* Mikania* species. In the present investigation, we aimed to evaluate 26 selected constituents of *Mikania* as potent inhibitory agents of human HMG-CoA reductase (hHMGR), human inducible nitric oxide synthase (hiNOS), and human squalene synthase (hSQS) using the in silico method.

Methodology

Twenty-six selected constituents of *Mikania* were investigated based on the docking behavior of three target enzymes, namely hHMGR, hiNOS, and hSQS, using the Cdocker method (Discovery Studio^®^ 3.1, Accelrys, Inc., San Diego, CA).

Results

Docking analysis showed that methyl-3,5-di-*O*-caffeoyl quinate (MCQ) has the maximum binding energy (BE) (-39.63, -50.65, and -58.56 kcal/mol) with hHMGR, hiNOS, and hSQS enzymes. On the other hand, six ligands (kaurenoic acid (KAA), stigmasterol (SS), grandifloric acid (GA), kaurenol (KA), spathlenol (SP), and taraxerol (TA)) of *Mikania* failed to dock with either of the target enzymes (hHMGR, hiNOS, or hSQS).

Conclusions

The findings of the current study provide new insight regarding 26 selected ligands of *Mikania* as potent inhibitory agents of hHMGR, hiNOS, and hSQS.

## Introduction

Members belonging to the genus *Mikania *are herbaceous weeds in nature and belong to the family Asteraceae (daisy) [1]. The genus *Mikania *was named after the Czech researcher Johann Christian Mikan to honor his scientific contributions [2].* Mikania micrantha*, an invasive species, was introduced into the north-eastern (NE) region of India during the Second World War period as a ground cover for tea plantations [3]. *Mikania *species is, moreover, commonly distributed in Assam, India and is a huge threat to tea cultivators [3]. Currently, *M. micrantha* has been reported in 15 Indian states, which include a World Wide Fund for Nature prime preservation site in the Western Ghats with different levels of invasion [4].

Several classes of phytoconstituents have been previously isolated and reported from different *Mikania *species, which are known to possess various biological activities. The major chemical classes of the *Mikania *genus include alkaloids, caffeoylquinic acid and derivatives, coumarins and derivatives, diterpenes, flavonoids, norhumulene-type sesquiterpenes [5], phenols, phytosterols, polyphenols, saponins, sesquiterpenes, sesquiterpenes lactones, tannins, and terpenoids [6-10]. Crude leaf and flower extracts of *M. micrantha* have been reported to inhibit cyclooxygenase (COX), lipoxygenase (LOX), inducible nitric oxide synthase (iNOS), myeloperoxidase, and protease enzyme activities [11]. Similarly, crude leaf and stem extracts of *M. micrantha* have been reported to inhibit angiotensin-1 converting enzyme (ACE), 3-hydroxy-3-methylglutaryl-coenzyme A reductase (HMGR), lipoprotein lipase (LPL), and pancreatic lipase (PL) enzyme activities [12]. Based on the above-mentioned information, we carried out the current work to analyze 26 chosen constituents (present in the whole plant, which are common among the *Mikania *species) of *Mikania *[6, 10] and aimed to study the docking analysis on human HMG-CoA reductase (hHMGR), human inducible nitric oxide synthase (hiNOS), and human squalene synthase (hSQS) by employing the Cdocker method.

## Materials and methods

Ligand preparation

Chemical structures of the 26 *Mikania *ligands were selected based on the literature search and current research interest and were retrieved from ChemSpider (www.chemspider.com), PubChem (www.pubchem.ncbi.nlm.nih.gov), and Chemical Entities of Biological Interest (ChEBI, www.ebi.ac.uk/chebi). They were as follows: a) mikamicranolide (MM) (ChemSpider ID: 10189069); b) kaurenoic acid (KAA) (PubChem ID: 73062); c) stigmasterol (SS) (PubChem ID: 5280794); d) grandifloric acid (GA) (PubChem ID: 159930); e) kaurenol (KA) (PubChem ID: 443465); f) spathulenol (SP) (PubChem ID: 522266); g) caryophyllene oxide (CO) (PubChem ID: 14350); h) syringaldehyde (SA)(PubChem ID: 8655); i) dihydrocoumarin (DC) (PubChem ID: 660); j) o-coumaric acid (CA) (ChemSpider ID: 553146); k) taraxerol (TA) (PubChem ID: 92097); l) melilotoside (ME) (PubChem ID: 5280759); m) patuletin (PA) (PubChem ID: 5281678); n) methyl-3,5-di-O-caffeoyl quinate (MCQ) (ChEBI ID: 66708); o) 3,3’,5-trihydroxy-4’,6,7-trimethoxyflavone (THTMF) (ChemSpider ID: 4476175’); p) psoralen (PS) (PubChem ID: 6199); q) curcumene (CU) (PubChem ID: 92139); r) herniarin (HE) (ChemSpider ID: 10295); s) bicyclogermacrene (BCG) (PubChem ID: 5315347); t) α-bisabolol (BI) (PubChem ID: 442343); u) γ-elemene (EL) (PubChem ID: 6432312); v) provincialin (PR)(ChEBI ID: 8599); w) dehydrocostus lactone (DHL) (PubChem ID: 73174); x) mikanin-3-O-sulfate (MS) (PubChem ID: 14630674) and y) nepetin (NE) (ChemSpider ID: 4476172). An unavailable three-dimensional (3D) structure of 2,6-dimethoxyquinone (DMQ) was prepared utilizing the ChemDraw software tool (Revvity Signals Software, Waltham, MA). All 26 ligands of *Mikania *were prepared in ChemDraw, and later molecular mechanics (MM2) minimization of ligands was performed by adopting the Chem3D software tool (CambridgeSoft Corporation, Cambridge, MA). Further, all the above-mentioned ligands were prepared using the Cdocker in-build ligand preparation procedure (Discovery Studio® 3.1, Accelrys, Inc., San Diego, CA) for the present study [1].

Target enzyme identification and preparation

Inflammation is the common pathway, where all three enzymes (hHMGR, hiNOS, and hSQS) are involved. More particularly, reactive oxygen species (ROS) and nitric oxide (NO) have been known to cause mitochondrial dysfunction, and previous reports have shown that cardiovascular (CV) disorders and metabolic syndromes (MetS) that are associated with cholesterol deregulation are related to mitochondrial damage [13]. Hence, the 3D structures of hHMGR (Protein Data Bank ID: 1DQ8 with a resolution of 2.10 Å), hiNOS (Protein Data Bank ID: 4NOS with a resolution of 2.25 Å), and hSQS (Protein Data Bank ID: 3ASX with 2.00 Å resolution) were downloaded from the Protein Data Bank (www.rcsb.org). “A” chain of all three enzymes was prepared separately by removing other chains (like B, C, and D), "ligand", and crystallographically observed "water". All the enzymes mentioned above were prepared using the Cdocker in-build protein preparation procedure (Accelrys, Inc.) [1].

Docking study

Docking analysis was performed for 26 chosen constituents of *Mikania *using the Cdocker standard procedure (under the Ligand-Protein Interaction Section). The Cdocker is a grid-based docking approach that uses Chemistry at HARvard Molecular Mechanics (CHARMM) force fields. For each selected ligand of Mikania, the 10 best "ligand binding poses" were ranked according to their Cdocker binding energies (BE). The binding interactions were predicted from the best among the 10 ligand binding poses, and finally, a standard procedure for in situ ligand minimization was employed [1].

## Results

The docking investigation and Cdocker BE analysis showed that MCQ exhibited the maximum interaction energy (-39.63 kcal per mol) with hHMGR. On the other hand, PS showed the least interaction energy (-13.65 kcal per mol) with the hHMGR enzyme. Three ligands (CA, MCQ, and HE) showed interaction with the Glu548 amino acid (AA) residue of hHMGR (Table 1). 

**Table 1:** Cdocker interaction energy analysis of 26 ligands with human HMG CoA reductase (hHMGR) using Discovery Studio® 3.1 F■: failed to dock; ◊: +-π interaction

Ligand	Cdocker interaction energy [-kcal/mol]	Interaction amino acid (AA) residue	Bond distance (Å)
Mikamicranolide (MM)	F^■^	-	-
Kaurenoic acid (KAA)	F^■^	-	-
Stigmasterol (SS)	F^■^	-	-
Grandifloric acid (GA)	F^■^	-	-
Kaurenol (KA)	F^■^	-	-
Spathulenol (SP)	F^■^	-	-
Caryophyllene oxide (CO)	F^■^	-	-
Syringaldehyde (SA)	F^■^	-	-
Dihydrocoumarin (DC)	F^■^	-	-
o-Coumaric acid (CA)	22.99	Glu548	2.0
Taraxerol (TA)	F^■^	-	-
Melilotoside (ME)	F^■^	-	-
Patuletin (PA)	F^■^	-	-
Methyl-3,5-di-O-caffeoyl quinate (MCQ)	39.63	Glu548	2.4
		Glu550	2.0
		Ser580	2.0
		Arg582^◊^	2.5
		Arg840	1.2
3,3’,5-Trihydroxy-4’,6,7-trimethoxyflavone (THTMF)	F^■^	-	-
Psoralen (PS)	13.65	No interaction	-
Curcumene (CU)	21.51	No interaction	-
Herniarin (HE)	18.14	Asp547	2.2
		Glu548	0.76 and 1.8
		Lys549	2.0
2,6-Dimethoxyquinone (DMQ)	F^■^	-	-
Bicyclogermacrene (BCG)	F^■^	-	-
α-Bisabolol (BI)	F^■^	-	-
γ-Elemene (EL)	F^■^	-	-
Provincialin (PR)	F^■^	-	-
Dehydrocostus lactone (DHL)	F^■^	-	-
Mikanin-3-O-sulfate (MS)	F^■^	-	-
Nepetin (NE)	F^■^	-	-

The docking investigation and Cdocker BE analysis showed that MCQ exhibited the highest interaction energy (-50.65 kcal per mol) with hiNOS. Whereas EL showed the lowest interaction energy (-12.26 kcal/mol) with hiNOS. Interestingly, 13 ligands (namely SA, DC, CA, ME, PA, MCQ, PS, CU, HE, DMQ, BI, PR, and MS) have shown interactions with the Trp372 AA residue of hiNOS, as shown in Table 2. 

**Table 2:** Cdocker interaction energy analysis of 26 ligands with human inducible nitric oxide synthase (hiNOS) using Discovery Studio® 3.1. F■: failed to dock; ◊- π- π: interaction; ▲: sigma-π interaction; ♦: π sigma interaction

Ligand	Cdocker interaction energy (-kcal/mol)	Interaction amino acid (AA) residue	Bond distance (Å)
Mikamicranolide (MM)	F^■^	-	-
Kaurenoic acid (KAA)	F^■^	-	-
Stigmasterol (SS)	F^■^	-	-
Grandifloric acid (GA)	F^■^	-	-
Kaurenol (KA)	F^■^	-	-
Spathulenol (SP)	F^■^	-	-
Caryophyllene oxide (CO)	F^■^	-	-
Syringaldehyde (SA)	30.57	Trp372	1.8 and 2.0
		Trp372^◊^	4.0
Dihydrocoumarin (DC)	23.05	Trp372	1.6
		Trp372^◊^	4.2
o-Coumaric acid (CA)	23.73	Trp372	2.0
Taraxerol (TA)	F^■^	-	-
Melilotoside (ME)	26.17	Trp372	2.1
		Ile433	1.8
		Met434^▲^	2.4
Patuletin (PA)	38.62	Trp194^◊^	4.6 and 4.7
		Ser242	1.2
		Phe369^◊^	4.7
		Asn370	1.7
		Trp372	2.4
Methyl-3,5-di-O-caffeoyl quinate (MCQ)	50.65	Gln205	1.1
		Tyr233	2.0, 2.1 and 2.4
		Ser242	1.4
		Trp372	2.3
		Trp372^◊^	4.9
3,3’,5-Trihydroxy-4’,6,7-trimethoxyflavone (THTMF)	38.50	No interaction	-
Psoralen (PS)	25.53	Trp372^◊^	4.2 and 4.7
Curcumene (CU)	23.59	Trp372^◊^	4.4
Herniarin (HE)	26.34	Trp372^◊^	4.8 and 5.8
2,6-Dimethoxyquinone (DMQ)	25.46	Trp372	1.7
Bicyclogermacrene (BCG)	12.85	No interaction	-
α-Bisabolol (BI)	26.55	Trp372	1.5
γ-Elemene (EL)	12.26	No interaction	-
Provincialin (PR)	37.28	Ile201	2.3
		Gly202	2.5
		Trp372^♦^	2.9
Dehydrocostus lactone (DHL)	F^■^	-	-
Mikanin-3-O-sulfate (MS)	39.74	Trp372	1.4 and 2.2
Nepetin (NE)	38.78	Glu377	0.94
		Ser442	2.4

A higher negative value represents the highest binding affinity of the ligand (a constituent), which shows the potent modulating effect against the target enzymes. In contrast, if a ligand (a constituent) exhibits minimum binding affinity or fails to dock, then it may possess weak or very weak biological activity.

Similarly, the docking investigation and Cdocker BE analysis showed that MCQ exhibited the maximum interaction energy (-58.56 kcal per mol) with hSQS, and DC showed the lowest interaction energy (-19.93 kcal/mol) with hSQS. Four ligands (MM, ME, MCQ, and THTMF) showed interactions with the Asn215 AA residue of hSQS (Table 3).

**Table 3:** Cdocker interaction energy analysis of 26 ligands with human squalene synthase (hSQS) using Discovery Studio® 3.1. F■: failed to dock; ◊: sigma-π interaction

Ligand	Cdocker interaction energy (-kcal/mol)	Interaction amino acid (AA) residue	Bond distance (Å)
Mikamicranolide (MM)	30.78	Asn215	2.0
Kaurenoic acid (KAA)	F^■^	-	-
Stigmasterol (SS)	F^■^	-	-
Grandifloric acid (GA)	F^■^	-	-
Kaurenol (KA)	F^■^	-	-
Spathulenol (SP)	F^■^	-	-
Caryophyllene oxide (CO)	25.28	No interaction	-
Syringaldehyde (SA)	27.36	Gln212	0.8
Dihydrocoumarin (DC)	19.93	No interaction	-
o-Coumaric acid (CA)	23.30	Ala176	1.8
		Gln212	1.5
Taraxerol (TA)	F^■^	-	-
Melilotoside (ME)	34.50	Asn215	1.8
		Gln293	1.5
Patuletin (PA)	38.02	Asp80	1.8 and 1.8
Methyl-3,5-di-O-caffeoyl quinate (MCQ)	58.56	Glu83	0.7
		Tyr191	1.8, 1.9 and 2.3
		Asn215	1.6 and 2.0
		Cys289	1.6
3,3’,5-Trihydroxy-4’,6,7-trimethoxyflavone (THTMF)	39.09	Gln212	1.9
		Asn215	2.0 and 2.3
Psoralen (PS)	20.86	Gln293	1.9
Curcumene (CU)	32.31	No interaction	-
Herniarin (HE)	25.12	No interaction	-
2,6-Dimethoxyquinone (DMQ)	20.92	No interaction	-
Bicyclogermacrene (BCG)	24.55	No interaction	-
α-Bisabolol (BI)	31.60	Gln212	1.4
γ-Elemene (EL)	27.48	No interaction	-
Provincialin (PR)	F^■^	-	-
Dehydrocostus lactone (DHL)	24.75	No interaction	-
Mikanin-3-O-sulfate (MS)	45.75	Asp80	2.0
		Val179^◊^	2.0
Nepetin (NE)	37.54	Asp80	1.7 and 1.8

## Discussion

The inhibition of HMGR enzyme activity is one of the new methods for managing hypercholesterolemia, including cardiovascular disease (CVD). Earlier, three phytoconstituents, namely curcumin from the *Curcuma longa* plant, docosanol from the *Saccharum arundinaceum* plant, and salvianolic acid C from the *Salvia miltiorrhiza* plant were demonstrated to suppress HMGR enzyme activity [14]. Recently, crude leaf and stem extracts of *M. micrantha* were reported to inhibit HMGR enzyme activity [12, 15]. However, in the current investigation, 21 ligands of Mikania, namely MM, KAA, SS, GA, KA, SP, CO, SA, DC, TA, ME, PA, THTMF, DMQ, BCG, BI, EL, PR, DHL, MS, and NE, failed to dock with hHMGR. Three ligands, CA, MCQ, and HE, have shown interaction with the Glu548 AA residue of hHMGR. The present finding was on par with an earlier report, where naringin, eriodictyol 7-glucuronide, isorohifolin, diosmin, rosmarinic acid, and menthoside were shown to interact with the Glu548 AA residue of hHMGR [16].

The crude extract of *Mikania laevigata* has been reported to inhibit nitric oxide production by inhibiting nitric oxide synthase activity [17]. Similarly, crude leaf and flower extracts of *M. micrantha* have been reported to inhibit iNOS enzyme activity [11]. However, in the present study, nine ligands namely, MM, KAA, SS, GA, KA, SP, CO, TA, and DHL, failed to dock with the hiNOS enzyme, which might be due to low binding processes [18]. Interestingly, 13 ligands of *Mikania, *SA, DC, CA, ME, PA, MCQ, PS, CU, HE, DMQ, BI, PR, and MS, have shown interactions with the Trp372 AA residue of hiNOS. The current finding was on par with an earlier report, where clinacoside C, shaftoside, and isoorientin were shown to interact with the Trp372 AA residue of hiNOS [19].

Inhibition of squalene synthase activity might lead to a decrease in circulating low-density lipoprotein (LDL) cholesterol levels by stimulating LDL receptors [20]. Chlorogenic acid (CA) from the *Prunus mume* plant has been demonstrated to suppress squalene synthase enzyme activity [21]. However, in the present study, seven ligands namely, KAA, SS, GA, KA, SP, TA, and PR, failed to dock with the hSQS enzyme, which might be due to low binding processes [18]. Interestingly, four ligands, MM, ME, MCQ, and THTMF, have shown interaction with the Asn215 AA residue of hSQS. The current finding was on par with earlier reports, where eriodictyol-7-glucuronide, luteolin-7-glucoside, rosmarinic acid, and cycloclinacoside A1 have been shown to interact with the Asn215 AA residue of hSQS [16, 19].

Limitations and future recommendations

The findings of the current study are based on in silico analysis, which provides new insight into these 26 ligands from *Mikania *against hHMGR, hiNOS, and hSQS enzyme inhibition activities. Furthermore, in vitro cytarabine (Ara-C)-resistant acute myeloid leukemia (AML) cell lines like THP1 and U937 are used for assaying hHMGR and hSQS inhibition activities. In the case of hiNOS inhibition activity, the U937 cell line is adopted, and in vivo (a Wistar rat model is used to assess the said enzyme activities under in vivo conditions), experiments are required to confirm these 26 ligands of *Mikania *as having potent inhibitory actions against hHMGR, hiNOS, and hSQS enzyme activities.

## Conclusions

The present study showed that six ligands of *Mikania *namely, KAA, SS, GA, KA, SP, and TA, failed to dock with all the target enzymes(hHMGR, hiNOS, and hSQS). Interestingly, MCQ showed the maximum BE (-39.63, -50.65, and -58.56 kcal/mol) with hHMGR, hiNOS, and hSQS enzymes, respectively. Thus, the results of the current study have shown new insight into these 26 ligands of *Mikania *as potent inhibitor agents against hHMGR, hiNOS, and hSQS concerning the treatment of hypercholesterolemia, including CVD.
